# Hybrid Neural Network Cerebellar Model Articulation Controller Design for Non-linear Dynamic Time-Varying Plants

**DOI:** 10.3389/fnins.2020.00695

**Published:** 2020-07-28

**Authors:** Tien-Loc Le, Tuan-Tu Huynh, Sung-Kyung Hong, Chih-Min Lin

**Affiliations:** ^1^Faculty of Mechanical and Aerospace, Sejong University, Seoul, South Korea; ^2^Department of Electrical Electronic and Mechanical Engineering, Lac Hong University, Bien Hoa, Vietnam; ^3^Department of Electrical Engineering, Yuan Ze University, Taoyuan, Taiwan

**Keywords:** neural network, cerebellar model articulation controller, time-varying plants, non-linear system, adaptive control

## Abstract

This study proposes a hybrid method to control dynamic time-varying plants that comprises a neural network controller and a cerebellar model articulation controller (CMAC). The neural-network controller reduces the range and quantity of the input. The cerebellar-model articulation controller is the main controller and is used to compute the final control output. The parameters for the structure of the proposed network are adjusted using adaptive laws, which are derived using the steepest-descent gradient approach and back-propagation algorithm. The Lyapunov stability theory is applied to guarantee system convergence. By using the proposed combination architecture, the designed CMAC structure is reduced, and it makes it easy to design the network size and the initial membership functions. Finally, numerical-simulation results demonstrate the effectiveness of the proposed method.

## Introduction

Nowadays, the control of non-linear systems is a topic that continues to attract many researchers because of its widespread applications. In many practical cases, the challenge of this topic is that its mathematical model is poorly known or uncertain (Liu et al., 2011). Furthermore, non-linear systems are susceptible to internal and external disturbances (Li et al., 2018). Therefore, in recent years, some studies have used neural networks (NNs) to approximate non-linear functions (Zhou and Zhang, 2015; Han, 2018). Some studies combined a neural network and other methods to achieve better control performance, such as proportional-integral-derivative (PID) NNs, fuzzy NNs, and sliding mode NNs (Zou et al., 2011; Zhou and Zhang, 2015; Lin and Le, 2017a; Zhao et al., 2018; Wang et al., 2019). Neural networks enable large-scale concurrent computing, processing, and adaptive weight adjustment, and they are simple and convenient (Prieto et al., 2016). Recently, many studies use NNs to address control problems, system identification and prediction problems. In 2013, Li et al. developed an optical-interference pattern-sensing method and neural-network classification for pretesting gap mura on thin-film transistor liquid crystal displays (Li et al., 2013). In 2017, Sun and Pan developed a reliable neural-network to control non-affine non-linear systems (Sun and Pan, 2017). In 2018, Wang et al. presented a memristor-based artificial neural network to predict house prices (Wang et al., 2018). However, neural networks require a considerable amount of computational resources, there is the risk of overfitting, and the architecture must be defined (Tu, 1996).

The concept of a cerebellar-model articulation controller (CMAC) was first proposed by Albus (1975). It is a type of neural network that uses a model of the mammalian cerebellum (associative memory). It addresses the problems of fast-growing size and the learning difficulties that are inherent to current neural networks. Several studies showed that, for applications that require online learning, CMACs perform better than simple neural networks (Lin and Chen, 2009; Guan et al., 2019). Since CMACs have a non-fully connected perceptron-like associative-memory network with overlapping receptive fields, they have fast learning performance, and its computation is simple. Contrarily, neural networks have a fully connected perceptron; therefore, all weights are updated during each learning cycle, so the learning capacity for a neural network is essentially global in nature and slow (Lin and Chen, 2009). The main advantages of CMACs over NNs, MLPs, and RBFNs are fast learning, simple computation, and good generalization capability (Lin et al., 2013). Recent studies have proposed some modified CMACs, such as function-link, self-organizing, and type-2 fuzzy CMACs that have better performance. In 2016, Lin et al. proposed a type-2 fuzzy CMAC for an adaptive filter (Lin et al., 2016). In 2017, Lin and Le used a wavelet CMAC to control non-linear systems (Lin and Le, 2017b). In 2018, Tsao et al. proposed the use of a deep CMAC for an adaptive noise-cancellation system (Tsao et al., 2018). A conventional CMAC also has some disadvantages, such as it is difficult to determine a suitable network size and to select the initial membership functions (MFs) to achieve the best performance (Lin and Chen, 2009). It is particularly difficult when the network has many inputs, and each input has a large range.

This study proposes a new method with a structure that includes a neural network connected in series with a CMAC. All inputs to the neural network reduce quantity and range. The outputs for the NN feed into the CMAC to compute the final outputs. This proposed network structure is referred to as a hybrid neural-network–CMAC (HNNCMAC). It is used to control dynamic time-varying plants. The motivation behind a cascade of two architectures was to allow for the inputs into the CMAC structure to be small, avoiding the difficulty in selecting a suitable network size and the initial membership functions. In the CMAC structure, the number of neurons in receptive-field spaces is increasing exponentially by the number of neurons in input space. Our proposed HNNCMAC controller using the NN to reduce the inputs for the CMAC, and then the structure of the modified CMAC in our proposed network will be smaller than the conventional CMAC. It is more effective when the number of inputs is large. In comparison with previous modified CMAC neural networks, as in Lin and Le (2017b) and Lin et al. (2018a,b), the proposed HNNCMAC has some advantages, such as small CMAC structure, and ease in designing network size and initial membership functions. The main contributions of this study are: (1) the successful design of an adaptive HNNCMAC system for the control of non-linear dynamic time-varying plants; (2) adaptive laws are derived using the steepest-descent gradient approach and a back-propagation algorithm; (3) input range and quantity in the proposed CMAC could be reduced by the NN pre-controller; (4) the stability of the proposed method is guaranteed by Lyapunov analysis; and (5) the method could be used for non-linear control problems, as proven by the results of numerical simulations.

The remaining sections of the paper are organized as follows. The design of the HNNCMAC is presented in section Methods. Section 3 presents the simulation results for controlling the dynamic time-varying plant. Section 4 provides the discussion. Finally, the conclusion is given in Section 5.

## Methods

### HNNCMAC Structure

**Figure 1:**
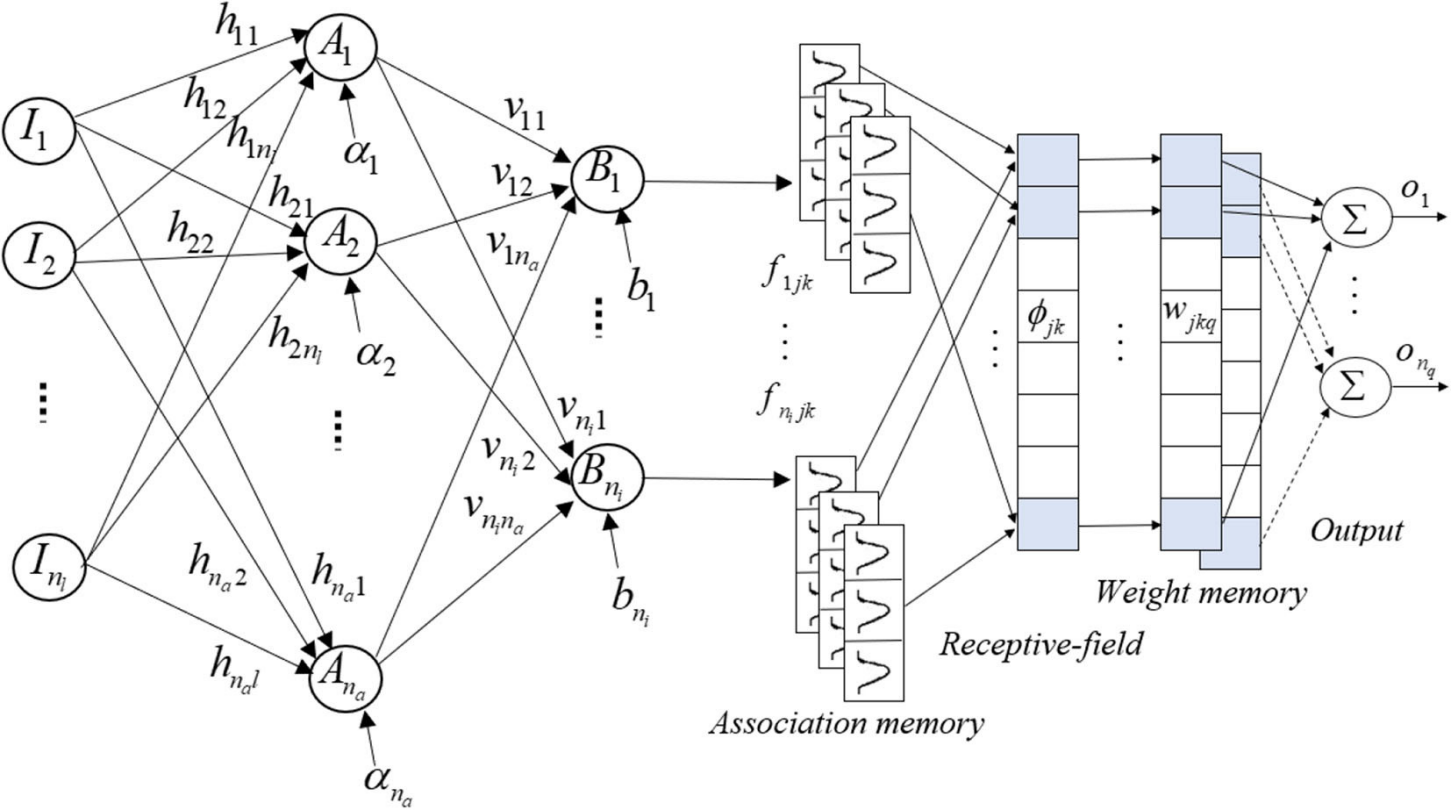
Hybrid neural-network–cerebellar-model articulation-controller structure.

The structure of hybrid NNCMAC includes a neural network that is connected in series with a CMAC. The NN reduces the range and the quantity of the input, and the output from the NN becomes the input for the CMAC to compute the final control output. Figure 1 shows the structure of HNNCMAC, which has seven spaces: input, hidden NN, output NN, association, receptive, weight-memory, and final-output spaces. These are described below.

(1) Input space *I*: There is no computation in this space. Input data from the dataset are fed into this space and directly transferred to the next space.

(2) Hidden NN space *A*: each node in this space performs a multiplication between vector input $I={\left[I_{1},\;I_{2},\ldots ,I_{n_{l}}\right]}^{T}$ and hidden NN weight matrix $h=\left[\begin{array}{l} h_{1}\\ h_{2}\\ \vdots \\ h_{n_{a}} \end{array}\right]=\left[\begin{array}{l} h_{11},h_{12},\ldots ,h_{1n_{l}}\\ h_{21},h_{22},\ldots ,h_{2n_{l}}\\ \vdots \;\ddots \;\vdots \\ h_{n_{a}1},\;h_{n_{a}2},\ldots ,h_{n_{a}n_{l}} \end{array}\right]$; after that, they are added with a bias $\alpha ={\left[\alpha_{1},\alpha_{2},\ldots ,\alpha_{n_{a}}\right]}^{T}$, where *n*_*a*_ is the number of nodes in the hidden NN space and *n*_*l*_ is the number of nodes in the input space; *h*_*al*_ is the connecting weight from the *a*^*th*^, *a* = 1, …, *n*_*a*_, neuron in space ***A*** to the *l*^*th*^, *l* = 1, …, *n*_*l*_ neuron in space ***I***.

For example, in this space, the output from the *x*^*th*^ node is derived as

(1) $$\begin{array}{r} A_{a}\;=\;\left[h_{a1},\;h_{a2},\ldots ,h_{an_{l}}\right]\left[\begin{array}{c} I_{1}\\ I_{2}\\ \ldots \\ I_{n_{l}} \end{array}\right]\;+\;\alpha_{x} \end{array}$$

where α_*a*_ is the bias of the *x*^*th*^ neuron.

Then, the output from this space is expressed as $A={\left[A_{1},A_{2},\ldots ,A_{n_{a}}\right]}^{T}$.

(3) Output NN space B: This is the output from the neural-network space and it is the input for the CMAC. This layer performs a multiplication between the vector in the previous layer $A={\left[A_{1},A_{2},\ldots ,A_{n_{a}}\right]}^{T}$ and output NN weight matrix $v=\left[\begin{array}{l} v_{1}\\ v_{2}\\ \vdots \\ v_{n_{i}} \end{array}\right]=\left[\begin{array}{l} v_{11},v_{12},\ldots ,v_{1n_{a}}\\ v_{21},v_{22},\ldots ,v_{2n_{a}}\\ \;\vdots \;\ddots \;\vdots \\ v_{n_{i}1},v_{n_{i}2},\ldots ,v_{n_{i}n_{a}} \end{array}\right]$; then, it adds to a bias $b={\left[b_{1},\;b_{2},\ldots ,b_{n_{i}}\right]}^{T}$. To limit the input range for the CMAC, the final result in this space is a tangent sigmoid function. The output for the *i*^*th*^ node is

(2) $$\begin{array}{r} B_{i}=\text{tansig}\left(net_{B_{i}}\right)\;=\;\frac{e^{net_{B_{i}}}-e^{-net_{B_{i}}}}{e^{net_{B_{i}}}+e^{-net_{B_{i}}}}\;fori=1,2,\ldots ,n_{i} \end{array}$$

where $net_{B_{i}}=\left[v_{i1},v_{i2},\ldots ,v_{in_{a}}\right]\left[\begin{array}{l} A_{1}\\ A_{2}\\ \ldots \\ A_{n_{a}} \end{array}\right]+b_{i}$; *v*_*ia*_ is the connecting weight from the *i*^*th*^, *i* = 1, …, *n*_*i*_, neuron in space ***B*** to the *a*^*th*^ neuron in space ***A***.

The output for this space is expressed as $B={\left[B_{1},B_{2},\ldots ,B_{n_{i}}\right]}^{T}$.

(4) Association space *F*: In this space, several elements are accumulated as a block. The membership grades in each block are calculated using input variables *B*_*i*_ from the previous space and the Gaussian MFs.

(3) $$\begin{array}{r} f_{ijk}=\text{exp}\left(-{\left(\frac{B_{i}-m_{ijk}}{\sigma_{ijk}}\right)}^{2}\right)\;for\;j=1,2,\ldots ,n_{j}\;and\\ k=1,2,\ldots ,n_{k} \end{array}$$

where *m*_*ijk*_ is the mean; σ_*ijk*_ is the variance of the *k*^*th*^ block in the *j*^*th*^ layer that corresponds to the *i*^*th*^ input variable; *n*_*j*_ is the number of layers; and *n*_*k*_ is the number of blocks. Therefore, the output from this space is the vector association.

(5) Receptive-field space ϕ : This layer performs the mapping that relates each location of *F* to generate the receptive-field vector:

$\phi ={\left[\phi_{11},...,\phi_{1n_{k}},...,\phi_{n_{j}1},...,\;\phi_{n_{j}n_{k}}\right]}^{T}\in {ℜ}^{n_{j}n_{k}}$

where

(4) $$\begin{array}{r} \phi_{jk}\;=\;\underset{i=1}{\overset{n_{i}}{\prod }}f_{ijk}\left(B_{i}\right) \end{array}$$

**Figure 2:**
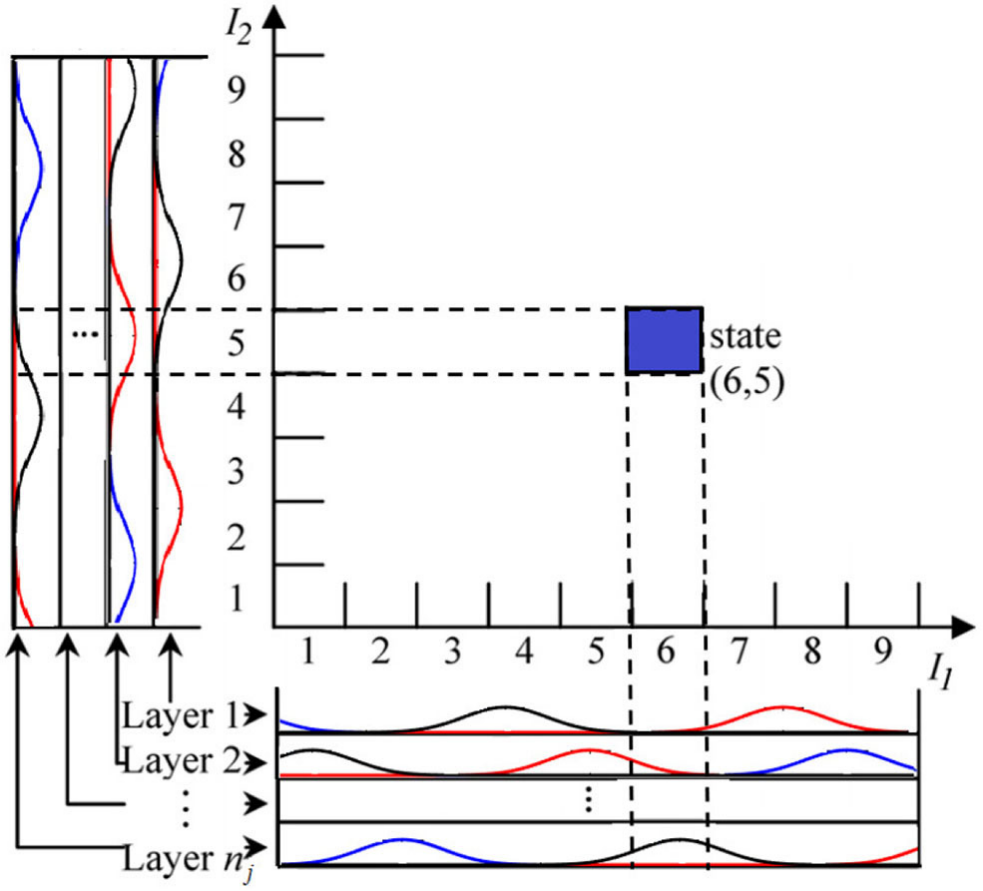
CMAC mapping with Gaussian membership function.

The mechanism for mapping 2D input is shown in Figure 2.

(6) Weight-memory space *W:* Each element of ϕ is mapped with a specific adjustable value for *W* that is expressed as

(5) $$\begin{array}{r} w_{jkq}\;=\;{\left[\begin{array}{ccc} w_{111},\ldots , & w_{1n_{k}1},...,w_{n_{j}11}, & ...,w_{n_{j}n_{k}1}\\ w_{112},..., & w_{1n_{k}2},...,w_{n_{j}12}, & ...,w_{n_{j}n_{k}2}\\ \vdots & \ddots & \vdots \\ w_{11n_{q}},..., & w_{1n_{k}n_{q}},...,w_{n_{j}1n_{q}}, & ...,w_{n_{j}n_{k}n_{q}} \end{array}\right]}^{T}\in {ℜ}^{n_{j}n_{k}n_{q}} \end{array}$$

where *w*_*jkq*_ is the connecting weight for the *q*th, *q* = 1, …, *n*_*q*_, final output and the receptive-field space for the *j*th layer and *k*th block.

(7) Final output space *O*: This space performs the product operation of receptive-field space ϕ and weight-memory space *W* to obtain the final output for the HNNCMAC, which is expressed as

(6) $$\begin{array}{r} u_{HNNCMAC}^{q}=o_{q}=w^{T}\phi =\overset{n_{j}}{\underset{j=1}{\sum }}\overset{n_{k}}{\underset{k=1}{\sum }}w_{jkq}\phi_{jk} \end{array}$$

The initial parameters for the HNNCMAC are chosen randomly and updated by some adaptive laws, which are derived using the steepest-descent gradient approach and a back-propagation algorithm, as described in the following section. The computational complexity using the Big-O notation is Big-O = O(T^*^[max(*p*_1_, *p*_2_, …, *p*_*n*_*j*__) +$\overset{n_{j}}{\underset{j=1}{\prod }}p_{j}$+*n*_*l*_*n*_*i*_*n*_*a*_]), where T is the running time, *p*_*j*_ is the number of membership functions in association space.

### HNNCMAC Parameters—Learning Algorithm

**Figure 3:**
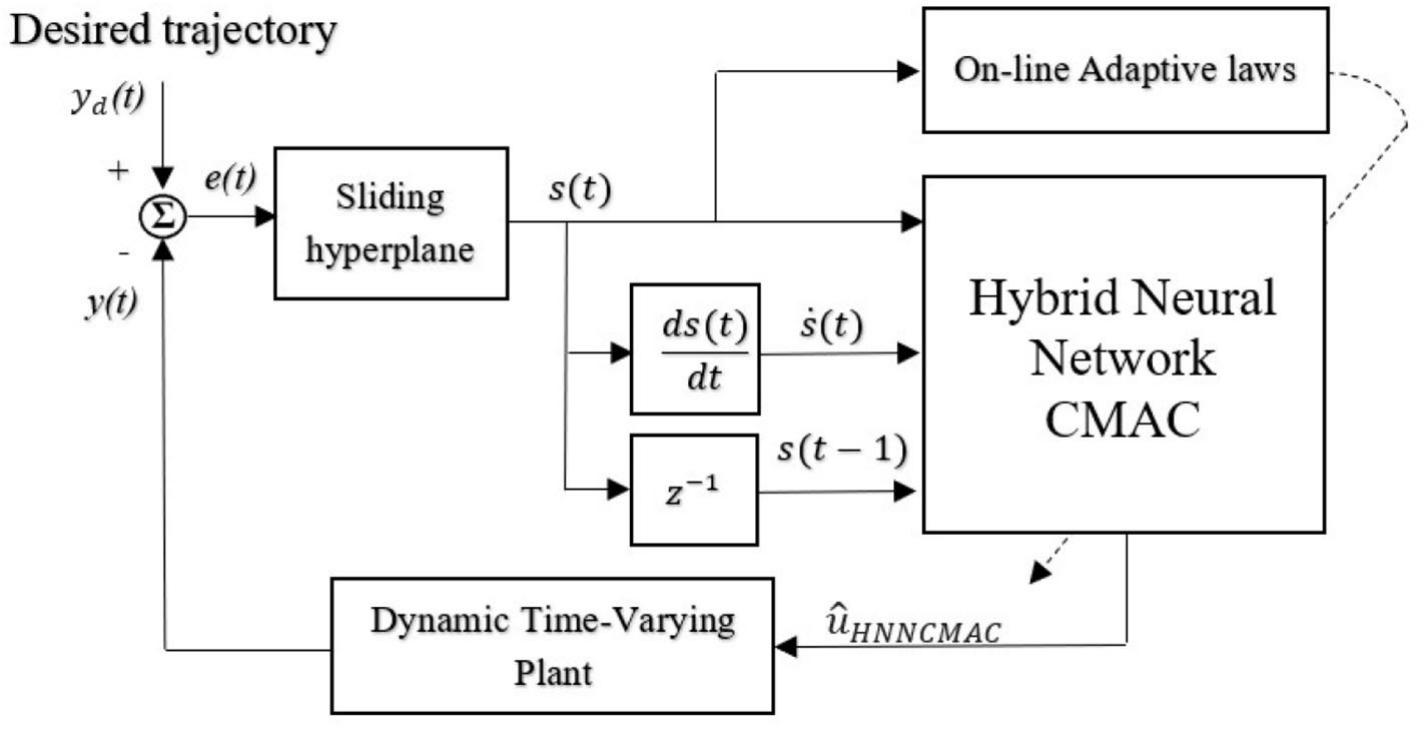
Scheme for HNNCMAC system.

**Figure 4:**
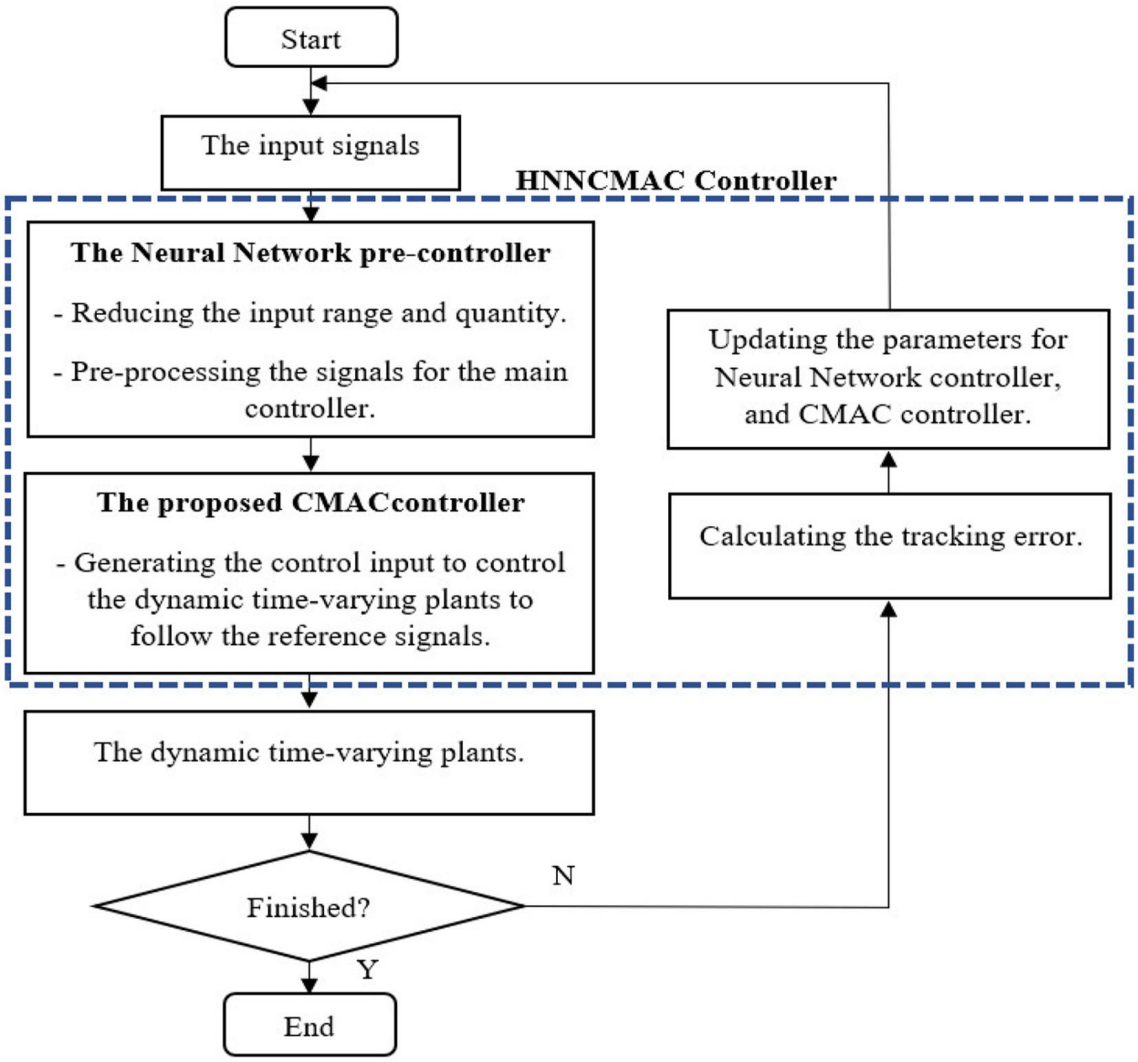
Flowchart of HNNCMAC system.

The scheme for the HNNCMAC system is shown in Figure 3. The goal of control system is to generate control signal û_*HNNCMAC*_(*t*), which forces the output of dynamic time-varying plant *y*(*t*) to track reference signal *y*_*d*_(*t*). The flowchart of the HNNCMAC system is shown in Figure 4, in which input range and quantity in the proposed CMAC could be reduced by the NN pre-controller. Therefore, it can reduce the number of neurons in receptive-field spaces and the weight-memory space; then, the structure of CMAC can be significantly reduced.

The high-order sliding mode from Manceur et al. (2012) and Zheng et al. (2014) is used to improve the performance of the control system

(7) $$\begin{array}{l} s\left(t\right)=\overset{n-1}{\underset{l=0}{\sum }}\frac{\left(n-1\right)!}{l!\left(n-l-1\right)!}{\left(\frac{\partial }{\partial t}\right)}^{n-l-1}\lambda^{l}e\\ \;=e^{\left(n-1\right)}+\left(n-1\right)\lambda e^{\left(n-2\right)}+\frac{\left(n-1\right)\left(n-2\right)}{2}\lambda^{2}e^{\left(n-3\right)}\\ \;\ldots \;+\;\lambda^{n-1}e \end{array}$$

where λ and *n* are the slope and the order of the sliding surface, respectively. Both λ and *n* are positive constants. Tracking error *e*(*t*) is defined as:

(8) $$\begin{array}{l} e\left(t\right)=y_{d}\left(t\right)-y\left(t\right)\in ℜ \end{array}$$

where *y*_*d*_ and *y* are reference signal and system output, respectively.

Taking the derivative of Equation (7)

(9) $$\begin{array}{l} ṡ\left(t\right)=e^{\left(n\right)}+\left(n-1\right)\lambda e^{\left(n-1\right)}+\frac{\left(n-1\right)\left(n-2\right)}{2}\lambda^{2}e^{\left(n-2\right)}\\ \;\ldots \;+\;\lambda^{n-1}e\\ \;=e^{\left(n\right)}+K^{T}e \end{array}$$

where $K={\left[\left(n-1\right)\lambda ,\frac{\left(n-1\right)\left(n-2\right)}{2}\lambda^{2},...,\lambda^{n-1}\right]}^{T}\in {ℜ}^{n-1}$ is the positive gain vector and $e\left(t\right)={\left[e^{\left(n-1\right)}\left(t\right),e^{\left(n-2\right)}\left(t\right),\ldots ,\dot{e}\left(t\right)\right]}^{T}\in {ℜ}^{n}$ is the tracking error vector.

If the values for *n* and λ correspond to the coefficients of a Hurwitz polynomial, then $\underset{k\to \infty }{\text{lim}}e\left(t\right)=0$.

The structure of the HNNCMAC has seven variables that are updated as: *w*_*jkq*_, *m*_*ijk*_, σ_*ijk*_, *b*_*i*_, *v*_*ia*_, α_*a*_ and *h*_*al*_. The Lyapunov cost function is chosen as $V\left(t\right)=\frac{1}{2}s^{2}\left(t\right)$, so $\dot{V}\left(t\right)=s\left(t\right)ṡ\left(t\right)$. An online learning gradient descent algorithm was used to minimize $\dot{V}\left(t\right)$. Therefore, online tuning laws for the HNNCMAC parameters are given by the following equations:

(10) $$\begin{array}{l} {ŵ}_{jkq}\left(k+1\right)={ŵ}_{jkq}\left(k\right)+\Delta {ŵ}_{jkq} \end{array}$$

(11) $$\begin{array}{l} {\hat{m}}_{ijk}\left(k+1\right)={\hat{m}}_{ijk}\left(k\right)+\Delta {\hat{m}}_{ijk} \end{array}$$

(12) $$\begin{array}{l} {\hat{\sigma }}_{ijk}\left(k+1\right)={\hat{\sigma }}_{ijk}\left(k\right)+\Delta {\hat{\sigma }}_{ijk} \end{array}$$

(13) $$\begin{array}{l} {\hat{b}}_{i}\left(k+1\right)={\hat{b}}_{i}\left(k\right)+\Delta {\hat{b}}_{i} \end{array}$$

(14) $$\begin{array}{l} {\hat{v}}_{ia}\left(k+1\right)={\hat{v}}_{ia}\left(k\right)+\Delta {\hat{v}}_{ia} \end{array}$$

(15) $$\begin{array}{l} {\hat{\alpha }}_{a}\left(k+1\right)={\hat{\alpha }}_{a}\left(k\right)+\Delta {\hat{\alpha }}_{a} \end{array}$$

(16) $$\begin{array}{l} {\hat{h}}_{al}\left(k+1\right)={\hat{h}}_{al}\left(k\right)+\Delta {\hat{h}}_{al} \end{array}$$

where ${ŵ}_{jkq},{\hat{m}}_{ijk},{\hat{\sigma }}_{ijk},{\hat{b}}_{i},{\hat{v}}_{ia},{\hat{\alpha }}_{a},{ĥ}_{al}$ are the estimation of the optimal values for parameters *w*_*jkq*_, *m*_*ijk*_, σ_*ijk*_, *b*_*i*_, *v*_*ia*_, α_*a*_, *h*_*al*_; and $\Delta {ŵ}_{jkq},\Delta {\hat{m}}_{ijk},\Delta {\hat{\sigma }}_{ijk},\Delta {\hat{b}}_{i},\Delta {\hat{v}}_{ia},\Delta {\hat{\alpha }}_{a},\Delta {ĥ}_{al}$ are the estimation of the optimal values for Δ*w*_*jkq*_, Δ*m*_*ijk*_, Δσ_*ijk*_, Δ*b*_*i*_, Δ*v*_*ia*_, Δα_*a*_, Δ*h*_*al*_.

The updating term in Equations (10–16) is obtained by back-propagation by using the following chain rules:

(17) $$\begin{array}{l} \Delta {ŵ}_{jkq}=-{\hat{\eta }}_{w}\frac{\partial \dot{V}\left(t\right)}{\partial {ŵ}_{jkq}}=-{\hat{\eta }}_{w}\frac{\partial \dot{V}\left(t\right)}{\partial {û}_{HNNCMAC}^{q}}\frac{\partial {û}_{HNNCMAC}^{q}}{\partial {ŵ}_{jkq}}={\hat{\eta }}_{w}s\left(t\right){\hat{\phi }}_{jk} \end{array}$$

(18) $$\begin{array}{l} \Delta {\hat{m}}_{ijk}=-{\hat{\eta }}_{m}\frac{\partial \dot{V}\left(t\right)}{\partial {\hat{m}}_{ijk}}=-{\hat{\eta }}_{m}\frac{\partial \dot{V}\left(t\right)}{\partial {û}_{HNNCMAC}^{q}}\frac{\partial {û}_{HNNCMAC}^{q}}{\partial {\hat{\phi }}_{jk}}\frac{\partial {\hat{\phi }}_{jk}}{\partial f_{ijk}}\frac{\partial f_{ijk}}{\partial {\hat{m}}_{ijk}}\\ \;=-{\hat{\eta }}_{m}s\left(t\right){ŵ}_{jkq}{\hat{\phi }}_{jk}\frac{2\left(B_{i}-{\hat{m}}_{ijk}\right)}{{\hat{\sigma }}_{ijk}^{2}} \end{array}$$

(19) $$\begin{array}{l} \Delta {\hat{\sigma }}_{ijk}=-{\hat{\eta }}_{\sigma }\frac{\partial \dot{V}\left(t\right)}{\partial {\hat{\sigma }}_{ijk}}=-{\hat{\eta }}_{\sigma }\frac{\partial \dot{V}\left(t\right)}{\partial {û}_{HNNCMAC}^{q}}\frac{\partial {û}_{HNNCMAC}^{q}}{\partial {\hat{\phi }}_{jk}}\frac{\partial {\hat{\phi }}_{jk}}{\partial f_{ijk}}\frac{\partial f_{ijk}}{\partial {\hat{\sigma }}_{ijk}}\\ \;=-{\hat{\eta }}_{\sigma }s\left(t\right){ŵ}_{jkq}{\hat{\phi }}_{jk}\frac{2{\left(B_{i}-{\hat{m}}_{ijk}\right)}^{2}}{{\hat{\sigma }}_{ijk}^{3}} \end{array}$$

(20) $$\begin{array}{l} \Delta {\hat{b}}_{i}=-{\hat{\eta }}_{b}\frac{\partial \dot{V}\left(t\right)}{\partial {\hat{b}}_{i}}\\ \;=-{\hat{\eta }}_{b}\frac{\partial \dot{V}\left(t\right)}{\partial {û}_{HNNCMAC}^{q}}\overset{n_{j}}{\underset{j=1}{\sum }}\overset{n_{k}}{\underset{k=1}{\sum }}\left(\frac{\partial {û}_{HNNCMAC}^{q}}{\partial {\hat{\phi }}_{jk}}\frac{\partial {\hat{\phi }}_{jk}}{\partial f_{ijk}}\frac{\partial f_{ijk}}{\partial B_{i}}\frac{\partial B_{i}}{\partial net_{B_{i}}}\frac{\partial net_{B_{i}}}{\partial {\hat{b}}_{i}}\right)\\ \;={\hat{\eta }}_{b}s\left(t\right)\overset{n_{j}}{\underset{j=1}{\sum }}\overset{n_{k}}{\underset{k=1}{\sum }}\left({ŵ}_{jkq}{\hat{\phi }}_{jk}\frac{2\left(B_{i}-{\hat{m}}_{ijk}\right)}{{\hat{\sigma }}_{ijk}^{2}}\left(1-B_{i}^{2}\right)\right) \end{array}$$

(21) $$\begin{array}{l} \Delta {\hat{v}}_{ia}=-{\hat{\eta }}_{v}\frac{\partial \dot{V}\left(t\right)}{\partial {\hat{v}}_{ia}}\\ \;=-{\hat{\eta }}_{v}\frac{\partial \dot{V}\left(t\right)}{\partial {û}_{HNNCMAC}^{q}}\overset{n_{j}}{\underset{j=1}{\sum }}\overset{n_{k}}{\underset{k=1}{\sum }}\left(\frac{\partial {û}_{HNNCMAC}^{q}}{\partial {\hat{\phi }}_{jk}}\frac{\partial {\hat{\phi }}_{jk}}{\partial f_{ijk}}\frac{\partial f_{ijk}}{\partial B_{i}}\frac{\partial B_{i}}{\partial net_{B_{i}}}\frac{\partial net_{B_{i}}}{\partial {\hat{v}}_{ia}}\right)\\ ={\hat{\eta }}_{v}s\left(t\right)\overset{n_{j}}{\underset{j=1}{\sum }}\overset{n_{k}}{\underset{k=1}{\sum }}\left({ŵ}_{jkq}{\hat{\phi }}_{jk}\frac{2\left(B_{i}-{\hat{m}}_{ijk}\right)}{{\hat{\sigma }}_{ijk}^{2}}\left(1-B_{i}^{2}\right)A_{i}\right) \end{array}$$

(22) $$\begin{array}{l} \Delta {\hat{\alpha }}_{a}=-{\hat{\eta }}_{a}\frac{\partial \dot{V}\left(t\right)}{\partial {\hat{\alpha }}_{a}}\\ \;=-{\hat{\eta }}_{a}\frac{\partial \dot{V}\left(t\right)}{\partial {û}_{HNNCMAC}^{q}}\overset{n_{j}}{\underset{j=1}{\sum }}\overset{n_{k}}{\underset{k=1}{\sum }}\left(\frac{\partial {û}_{HNNCMAC}^{q}}{\partial {\hat{\phi }}_{jk}}\frac{\partial {\hat{\phi }}_{jk}}{\partial f_{ijk}}\frac{\partial f_{ijk}}{\partial B_{i}}\frac{\partial B_{i}}{\partial net_{B_{i}}}\right)\\ \;\left(\left(\overset{n_{i}}{\underset{i=1}{\sum }}\frac{\partial net_{B_{i}}}{\partial A_{a}}\right)\frac{\partial A_{a}}{\partial {\hat{\alpha }}_{a}}\right)\\ \;={\hat{\eta }}_{a}s\left(t\right)\overset{n_{j}}{\underset{j=1}{\sum }}\overset{n_{k}}{\underset{k=1}{\sum }}\left({ŵ}_{jkq}{\hat{\phi }}_{jk}\frac{2\left(B_{i}-{\hat{m}}_{ijk}\right)}{{\hat{\sigma }}_{ijk}^{2}}\left(1-B_{i}^{2}\right)\left(\overset{n_{i}}{\underset{i=1}{\sum }}v_{ia}\right)\right) \end{array}$$

(23) $$\begin{array}{l} \Delta {ĥ}_{al}=-{\hat{\eta }}_{h}\frac{\partial \dot{V}\left(t\right)}{\partial {ĥ}_{al}}\\ \;=-{\hat{\eta }}_{h}\frac{\partial \dot{V}\left(t\right)}{\partial {û}_{HNNCMAC}^{q}}\overset{n_{j}}{\underset{j=1}{\sum }}\overset{n_{k}}{\underset{k=1}{\sum }}\left(\frac{\partial {û}_{HNNCMAC}^{q}}{\partial {\hat{\phi }}_{jk}}\frac{\partial {\hat{\phi }}_{jk}}{\partial f_{ijk}}\frac{\partial f_{ijk}}{\partial B_{i}}\frac{\partial B_{i}}{\partial net_{B_{i}}}\right)\\ \;\left(\left(\overset{n_{i}}{\underset{i=1}{\sum }}\frac{\partial net_{B_{i}}}{\partial A_{a}}\right)\frac{\partial A_{a}}{\partial {ĥ}_{al}}\right)\\ \;={\hat{\eta }}_{h}s\left(t\right)\overset{n_{j}}{\underset{j=1}{\sum }}\overset{n_{k}}{\underset{k=1}{\sum }}\left({ŵ}_{jkq}{\hat{\phi }}_{jk}\frac{2\left(B_{i}-{\hat{m}}_{ijk}\right)}{{\hat{\sigma }}_{ijk}^{2}}\left(1-B_{i}^{2}\right)\left(\overset{n_{i}}{\underset{i=1}{\sum }}v_{ia}\right)I_{l}\right) \end{array}$$

where ${\hat{\eta }}_{m},{\hat{\eta }}_{\sigma },{\hat{\eta }}_{b},{\hat{\eta }}_{v},\;{\hat{\eta }}_{a},{\hat{\eta }}_{h}$ are the positive learning rates for the adaptive laws.

Using this online tuning parameter, the HNNCMAC can adjust the parameters online to achieve desired performance.

Proof of the algorithm convergence:

The Lyapunov cost function is defined as

(24) $$\begin{array}{l} V\left(t\right)=\frac{1}{2}s^{2}\left(t\right) \end{array}$$

Therefore, the rate of change for Equation (24) is

(25) $$\begin{array}{l} \Delta V\left(t\right)=V\left(t+1\right)-V\left(t\right)=\frac{1}{2}\left[s^{2}\left(t+1\right)-s^{2}\left(t\right)\right] \end{array}$$

By using the Taylor expansion, the difference in the sliding hyperplane is

(26) $$\begin{array}{l} s\left(t+1\right)=s\left(t\right)+\Delta s\left(t\right)≅s\left(t\right)+\left[\frac{\partial s\left(t\right)}{\partial {ŵ}_{jkq}}\right]\Delta {ŵ}_{jkq} \end{array}$$

From Equation (17), it can be seen that

(27) $$\begin{array}{l} \frac{\partial s\left(t\right)}{\partial {ŵ}_{jkq}}=-{\hat{\phi }}_{jk}≜\xi \end{array}$$

By using Equations (27) and (17), Equation (26) is rewritten as

(28) $$\begin{array}{l} s\left(t+1\right)=s\left(t\right)-\xi \left({\hat{\eta }}_{w}s\left(t\right)\xi \right)=s\left(t\right)\left[1-{\hat{\eta }}_{w}\xi^{2}\right] \end{array}$$

By using Equation (28), Equation (25) is rewritten as

(29) $$\begin{array}{l} \Delta V\left(t\right)=\frac{1}{2}s^{2}\left(t\right)\left[{\left(1-\eta_{w}\xi^{2}\right)}^{2}-1\right]\\ \;=\frac{1}{2}s^{2}\left(t\right)\left[{\left({\hat{\eta }}_{w}\xi^{2}\right)}^{2}-2{\hat{\eta }}_{w}\xi^{2}\right]\\ \;=\frac{1}{2}{\hat{\eta }}_{w}s^{2}\left(t\right)\xi^{2}\left({\hat{\eta }}_{w}\xi^{2}-2\right) \end{array}$$

From Equation (29), if the learning rate ${\hat{\eta }}_{w}$ is $0<{\hat{\eta }}_{w}<\frac{2}{\xi^{2}}$, then term Δ*V*(*t*) is negative, and Lyapunov function *V*(*t*) > 0. Therefore, the convergence of the system is guaranteed by Lyapunov stability. A similar method is used to prove the stability of learning rates ${\hat{\eta }}_{m},{\hat{\eta }}_{\sigma },{\hat{\eta }}_{b},{\hat{\eta }}_{v},\;{\hat{\eta }}_{a},{\hat{\eta }}_{h}$.

## Simulation of Results

In this section, the performance of the proposed HNNCMAC is investigated. Three examples in control of the dynamic time-varying plants are considered. The dynamic time-varying plants are the plants that contain the parameters varying with time.

**Example 1:** Controlling a dynamic time-varying plant borrowed from Narendra and Parthasarathy (1990) and Abiyev and Kaynak (2010), which is described by the difference equation

(30) $$\begin{array}{l} y\left(t\right)=f\left[y\left(t-1\right),y\left(t-2\right)\right]+u\left(t\right)+\varepsilon \left(t\right)+\Delta y\left(t\right) \end{array}$$

where *u(t)* is the control signal from the proposed HNNCMAC; *y(t), y(t-1)*, and *y(t-2)* are measurable plant output, one-step delayed plant output, and two-step delayed plant output, respectively; ε(*t*) = 0.1 sin(π*t*) and Δ*y*(*t*) = 0.1 *y*(*t*), respectively, denote the external disturbances and the system uncertainties; *f*[*y*(*t* − 1), *y*(*t* − 2)] is the previous plant output function, which is given as $f\left[y\left(t-1\right),y\left(t-2\right)\right]=\frac{y\left(t-1\right)y\left(t-2\right)\left(y\left(t-1\right)+2.5\right)}{\left(1+y{\left(t-1\right)}^{2}+y{\left(t-2\right)}^{2}\right)}$

The desired trajectory signal *y*_*d*_(*t*) is given as

(31) $$\begin{array}{l} y_{d}\left(t\right)=\{\begin{array}{cc} 10, & 0<t\leq 50\\ 15, & 50<t\leq 100\\ 10, & 100<t\leq 150\\ 15, & t>150 \end{array} \end{array}$$

**Figure 5:**
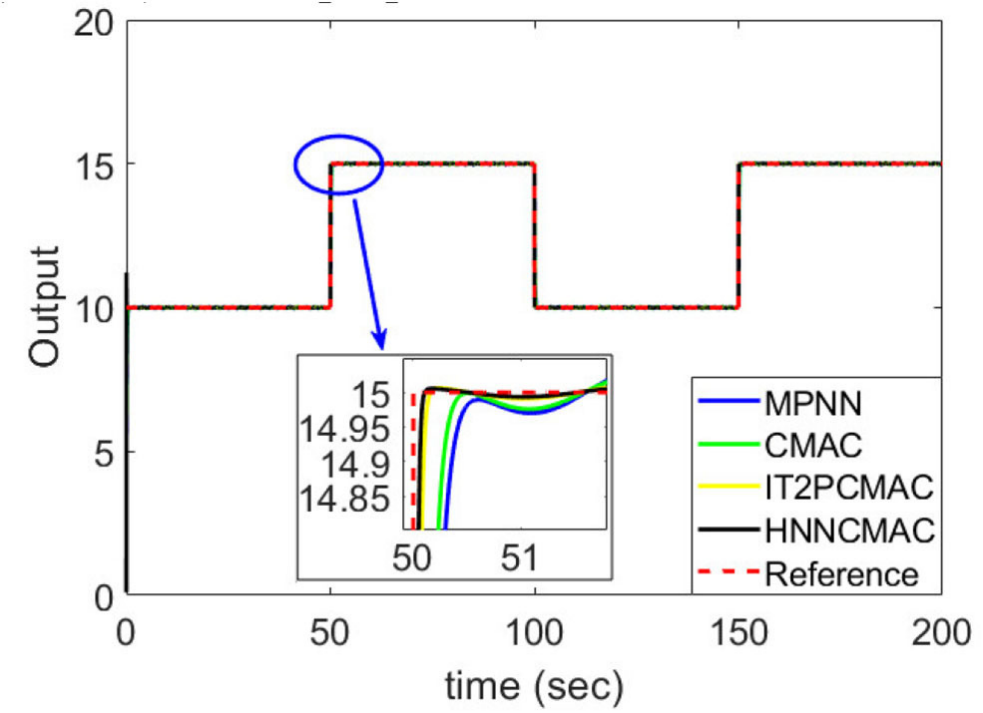
Comparison of system outputs between proposed HNNCMAC and other control methods for Example 1.

**Figure 6:**
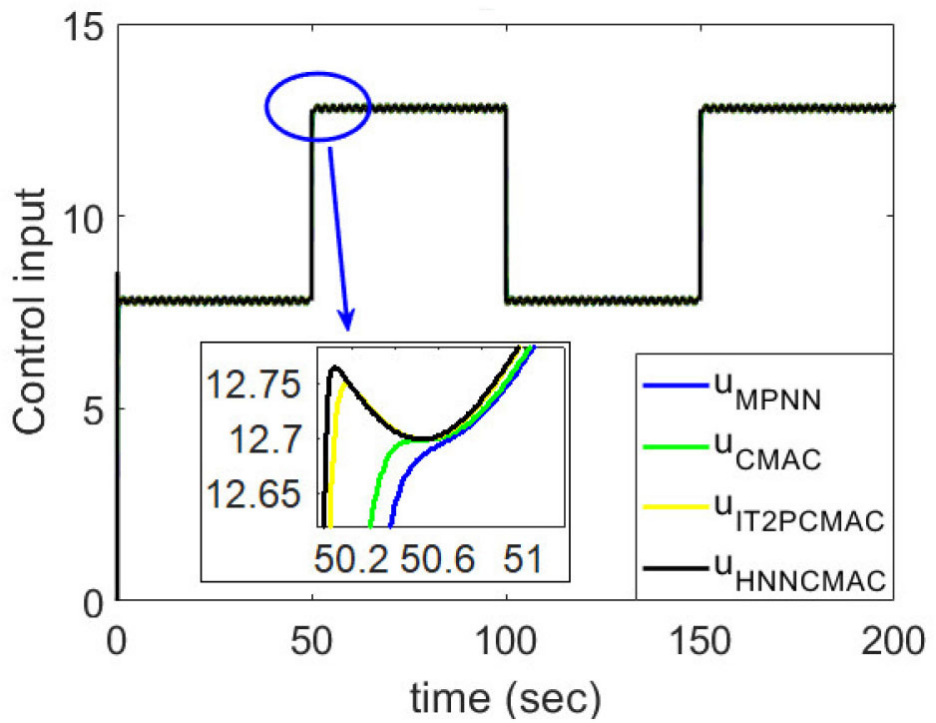
Comparision of control signals between proposed HNNCMAC and other control methods for Example 1.

**Figure 7:**
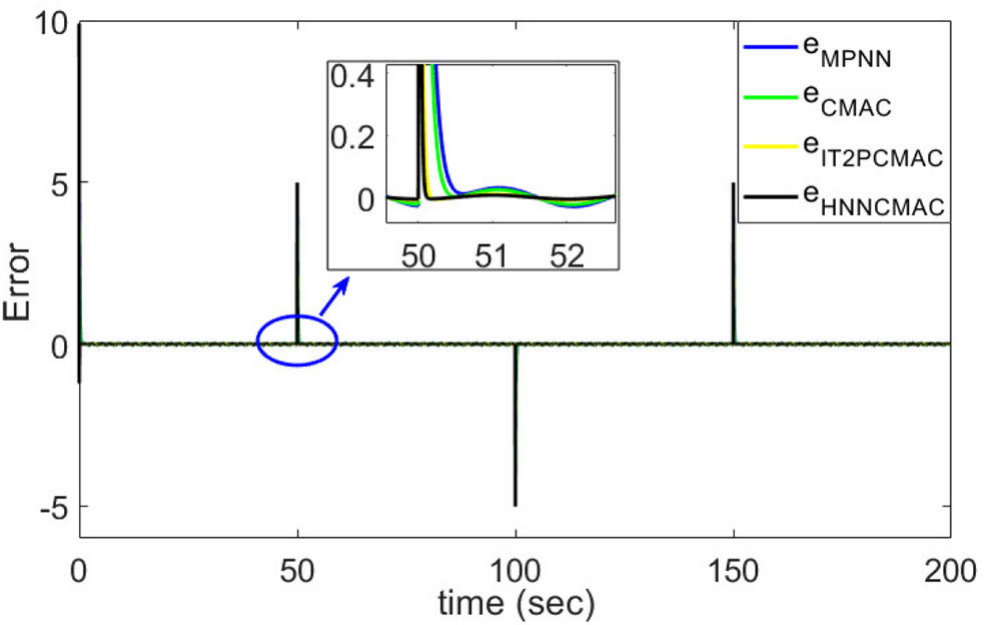
Comparison of tracking errors between proposed HNNCMAC and other control methods for Example 1.

The desired trajectory signal and the system outputs for the dynamic time-varying plant are shown in Figure 5. Control signals and tracking errors are shown in Figures 6, 7, respectively. These results show that the HNNCMAC allows a time-varying plant to follow a specified trajectory signal. In terms of the performance of the control system, Table 1 shows a comparison of the root mean square error (RMSE) for the proposed method and other methods.

**Table 1:** Comparison results in root mean square error (RMSE) of control time-varying systems.

**Control method**	**Computation time (s)**	**Example 1**	**Example 2**	**Example 3 (Square)**	**Example 3 (Sinusoidal)**
MPNN	0.0158	0.1878	0.8679	1.7629	0.4901
Conventional CMAC	0.0327	0.1692	0.8407	1.7141	0.4552
T2TSKFNS	0.0416	0.1469	0.7395	*N*	*N*
IT2PCMAC	0.0382	0.1408	0.7683	1.5297	0.4225
HNNCMAC (proposed controller)	0.0254	0.1215	0.6708	1.1644	0.3498

*N: the articles did not show those results. Note: MPNN, multilayer perceptron neural network; T2TSKFNS, Takagi–Sugeno–Kang fuzzy neural system; IT2PCMAC, interval type-2 Petri CMAC*.

**Example 2**: Controlling a dynamic time-varying plant borrowed from Zhang et al. (1998) and Abiyev and Kaynak (2010), which is described by the difference equation

(32) $$\begin{array}{l} y\left(t\right)=f\left[y\left(t-1\right),y\left(t-2\right)\right]+b_{0}\left(t\right)u\left(t\right)+\varepsilon \left(t\right)+\Delta y\left(t\right) \end{array}$$

where *u(t)* is the control signal from the proposed HNNCMAC; *y(t), y(t-1)*, and *y(t-2)* are measurable plant output, one-step delayed plant output, and two-step delayed plant output, respectively; ε(*t*) = 0.1 sin(π*t*) and Δ*y*(*t*) = 0.1 *y*(*t*), respectively, denote the external disturbances and the system uncertainties; *f*[*y*(*t* − 1), *y*(*t* − 2)] is the previous plant output function, which is given as *f*[*y*(*t* − 1), *y*(*t* − 2)] = *b*_1_(*t*)*y*(*t* − 1)+*b*_2_(*t*)*y*(*t* − 2); *b*_0_(*t*), *b*_1_(*t*), and *b*_2_(*t*) are the time-varying function, which are given as $b_{0}\left(t\right)=-\frac{t^{2}}{1+a_{1}\left(t\right)t+a_{2}\left(t\right)t^{2}}$; $b_{1}\left(t\right)=\frac{2+a_{1}\left(t\right)t}{1+a_{1}\left(t\right)t+a_{2}\left(t\right)t^{2}}$; $b_{2}\left(t\right)=-\frac{1}{1+a_{1}\left(t\right)t+a_{2}\left(t\right)t^{2}}$; *a*_1_(*t*) and *a*_2_(*t*) are the time-varying plant parameters, which are given as

(33) $$\begin{array}{l} a_{1}\left(t\right)=\frac{0.1t}{t+1};a_{2}\left(t\right)=\{\begin{array}{cc} 0.3, & 0\leq t<40\\ 0.1, & 40\leq t<60\\ 0.6, & 60\leq t<85\\ 0.3, & t>85 \end{array} \end{array}$$

The desired trajectory signal is given as

(34) $$\begin{array}{l} y_{d}\left(t\right)=\{\begin{array}{cc} 10, & 0<t\leq 25\\ 15, & 25<t\leq 50\\ 10, & 50<t\leq 75\\ 15, & t>75 \end{array} \end{array}$$

**Figure 8:**
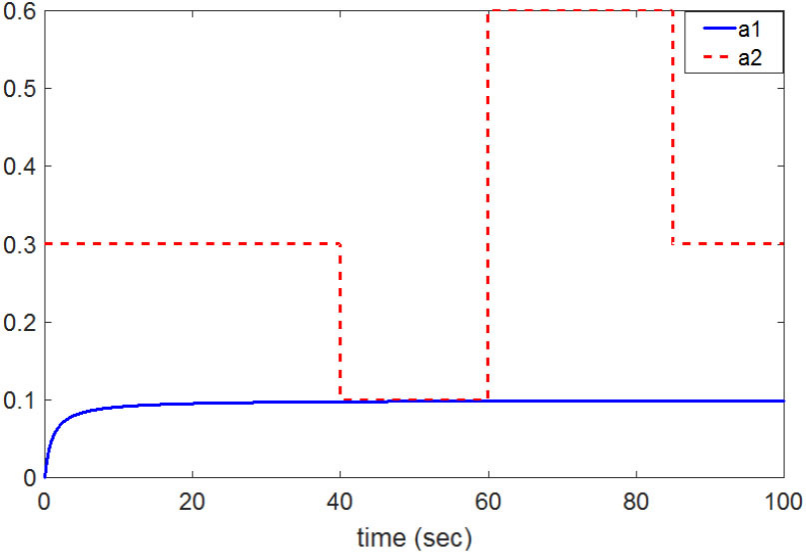
Change of time-varying parameters *a*_1_ and *a*_2_.

**Figure 9:**
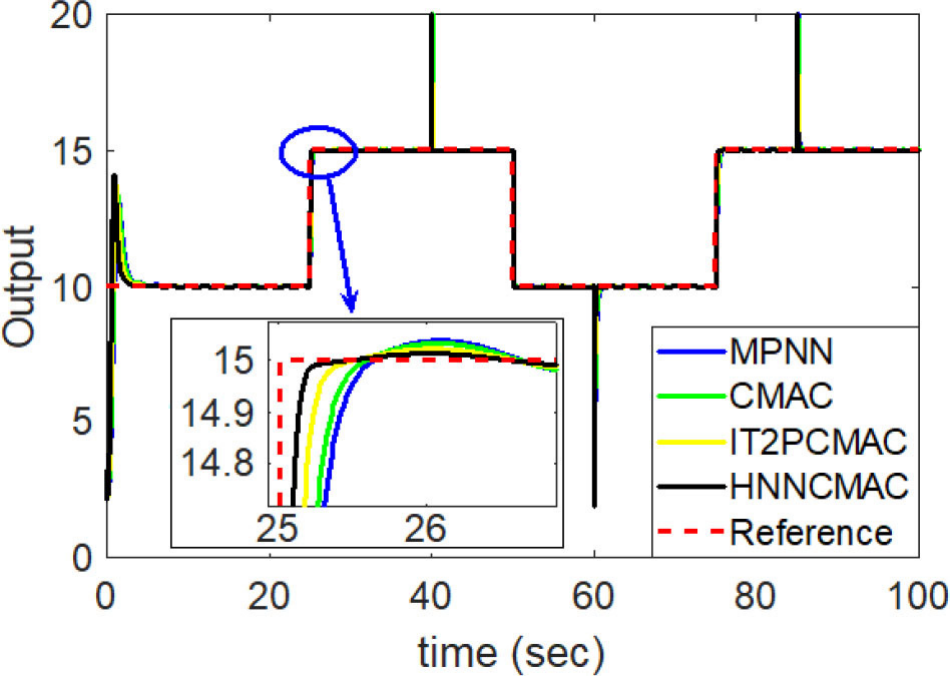
Comparison of system outputs between proposed HNNCMAC and other control methods for Example 2.

**Figure 10:**
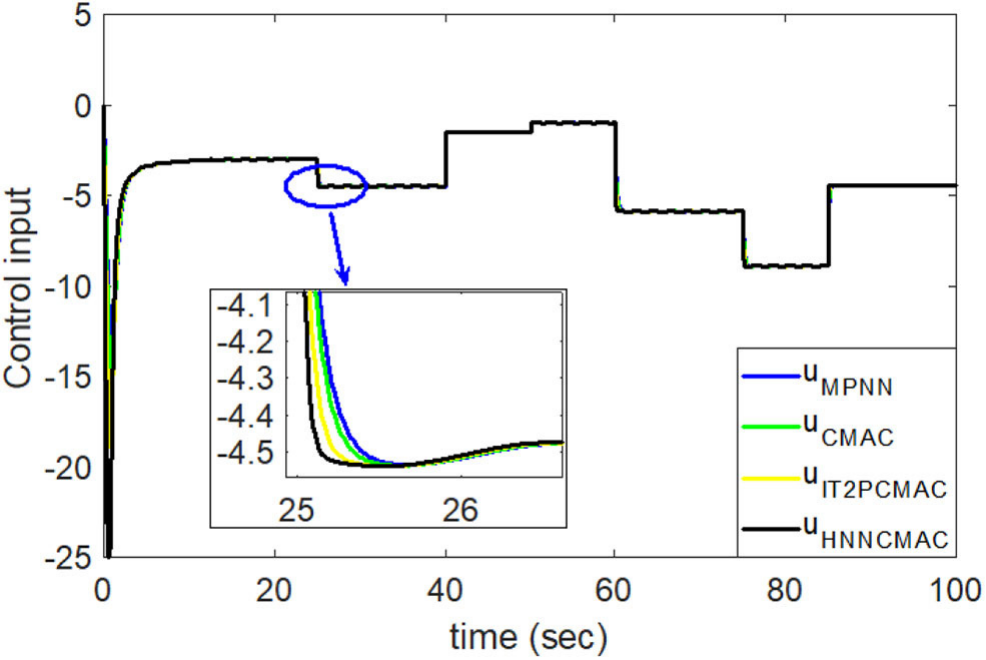
Comparision of control signals between proposed HNNCMAC and other control methods for Example 2.

**Figure 11:**
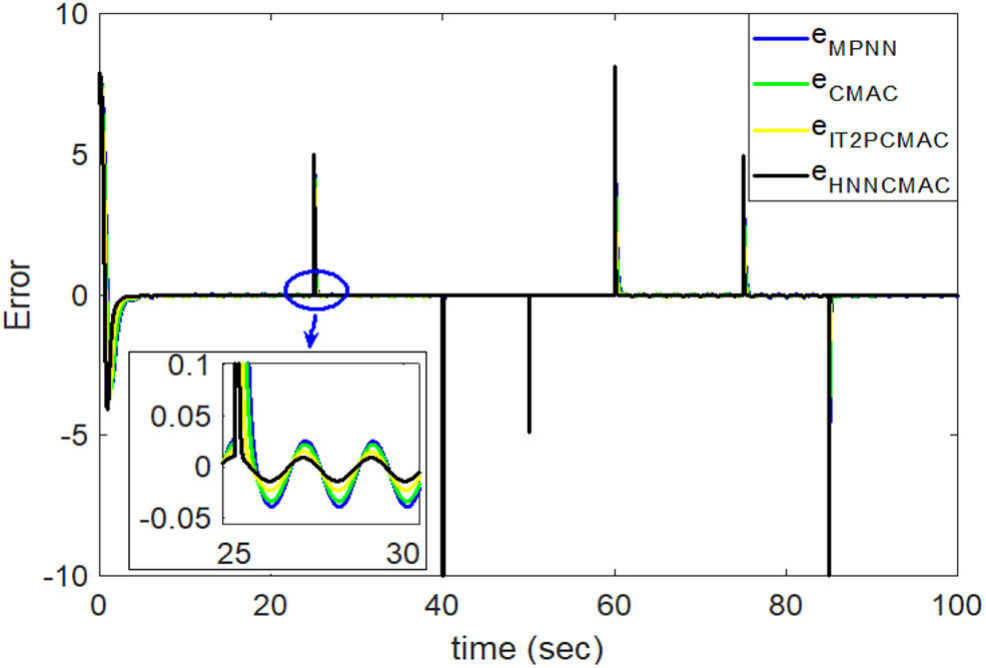
Comparison of tracking errors between proposed HNNCMAC and other control methods for Example 2.

Figure 8 shows the change in the time-varying parameters. The desired trajectory signal and the outputs for the dynamic time-varying plant are shown in Figure 9. Control signals and tracking errors are shown in Figures 10, 11, respectively. Simulation results showed that the HNNCMAC allows a time-varying plant to follow the reference signal, even if there are abrupt changes in parameters *a*_1_ and *a*_2_. Table 1 shows a comparison of the RMSE for the proposed method and other methods.

**Example 3**: Controlling a dynamic time-varying plant to follow variable frequency signals.

This example uses the same dynamic time-varying plant that is described in Example 2. The desired trajectory signal is the variable frequency signal:

(35) $$\begin{array}{l} y_{d1}\left(t\right)=5*square\left(2\pi tk_{t}\right) \end{array}$$

and

(36) $$\begin{array}{l} y_{d2}\left(t\right)=5*sin\left(2\pi tk_{t}\right) \end{array}$$

where *square* and *sin* are the square function and the sinusoidal function, respectively, and *k*_*t*_ is the parameter for changing the signal frequency, which changes by time as follows:

(37) $$\begin{array}{l} k_{t}\left(t\right)=\{\begin{array}{cc} 0.1, & 0\leq t<40\\ 0.5, & 40\leq t<60\\ 0.75, & 60\leq t<85\\ 1.0, & t>85 \end{array} \end{array}$$

**Figure 12:**
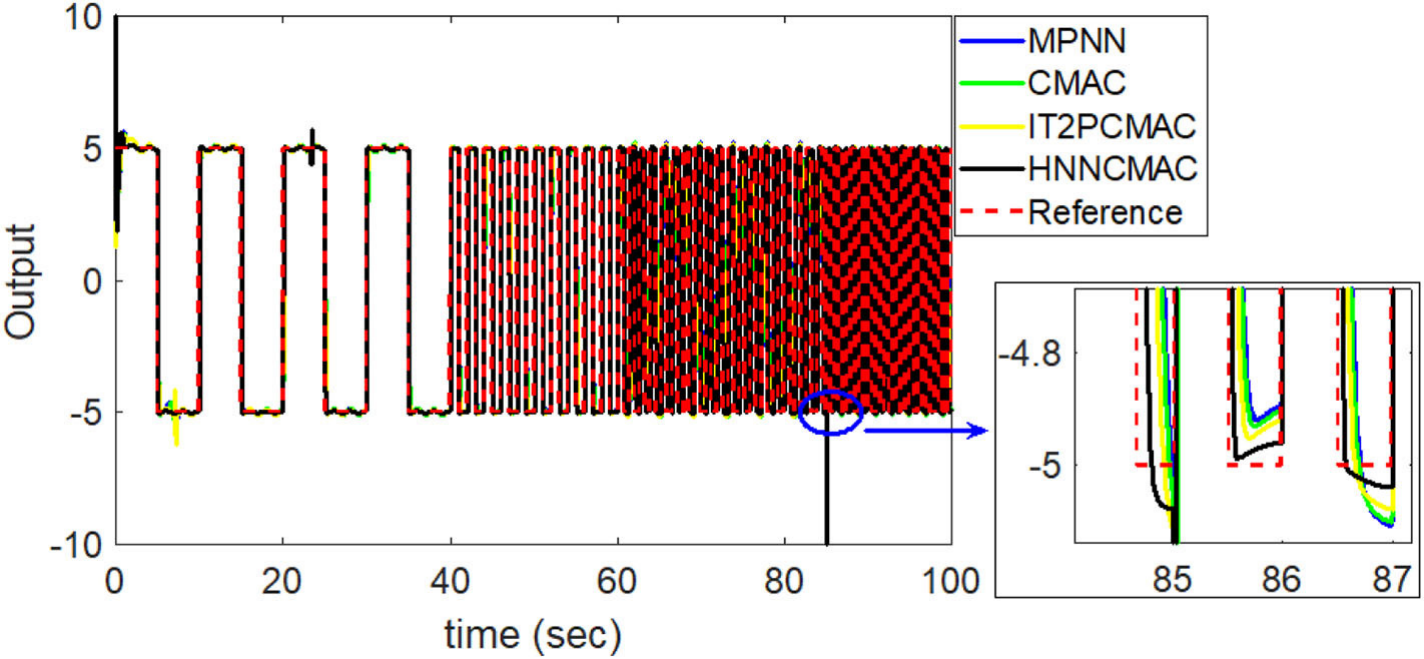
Comparison of system outputs between proposed HNNCMAC and other control methods for square signal reference with varying frequency.

**Figure 13:**
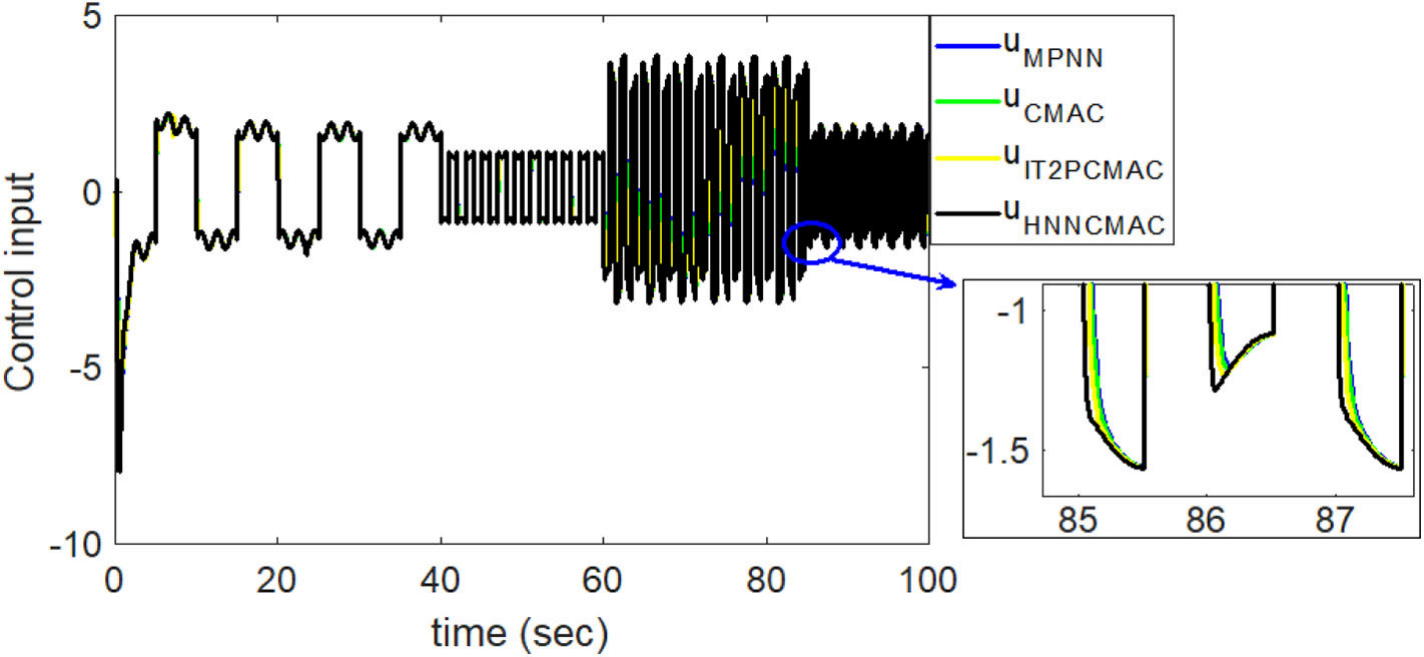
Comparision of control signals between proposed HNNCMAC and other control methods for square signal reference with varying frequency.

**Figure 14:**
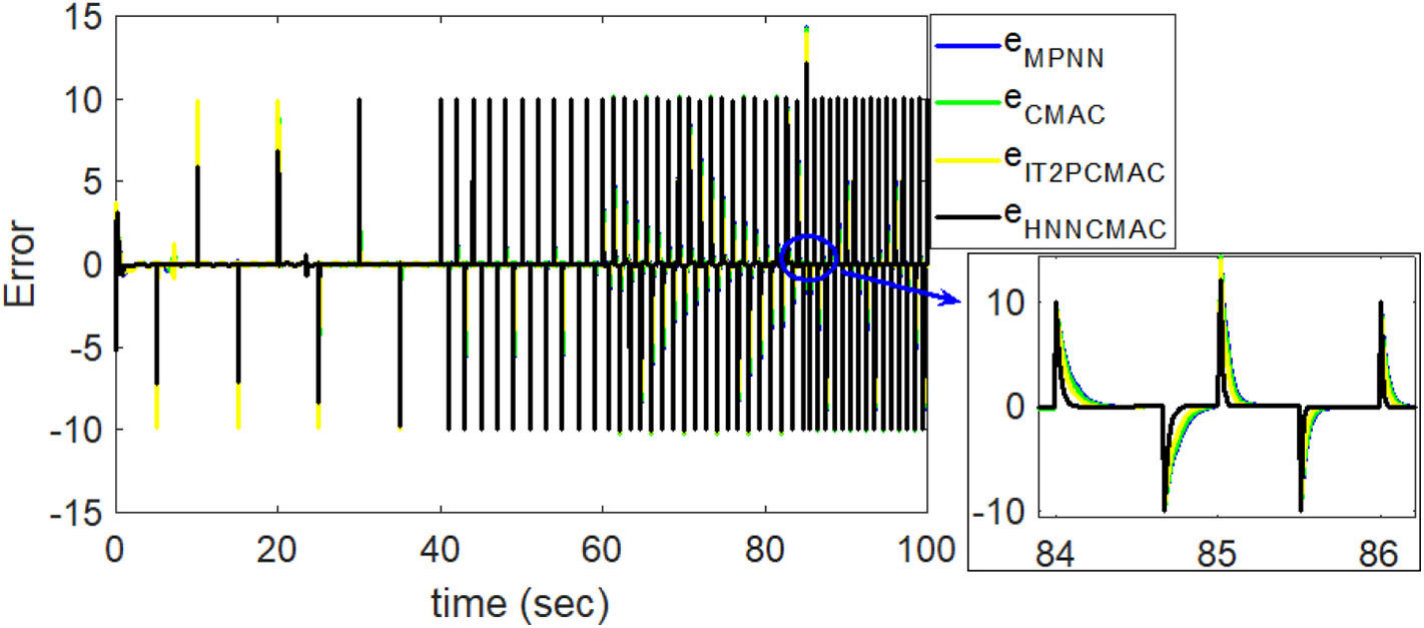
Comparison of tracking errors between proposed HNNCMAC and other control methods for square signal reference with varying frequency.

**Figure 15:**
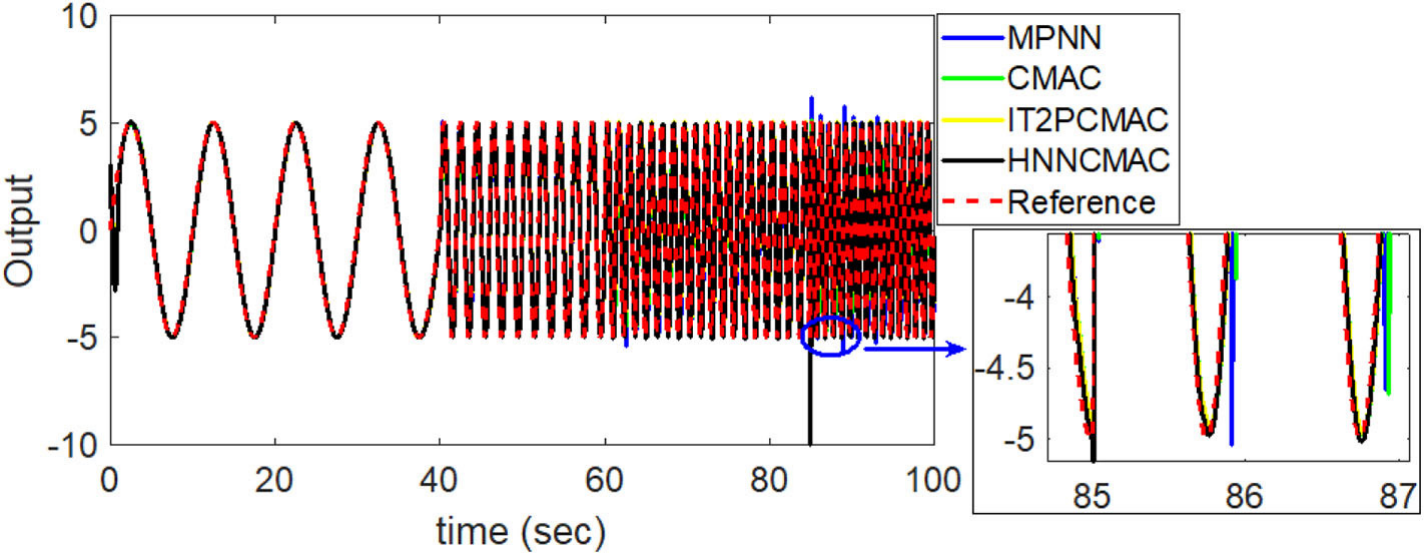
Comparison of system outputs between proposed HNNCMAC and other control methods for sinusoidal signal reference with varying frequency.

**Figure 16:**
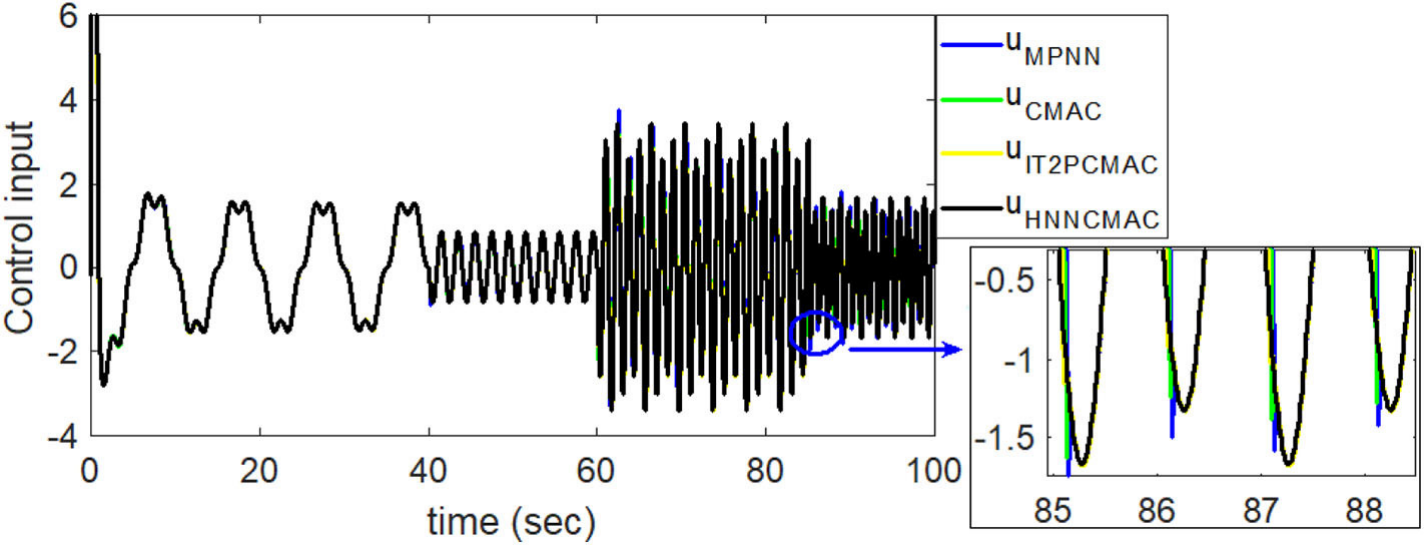
Comparision of control signals between proposed HNNCMAC and other control methods for sinusoidal signal reference with varying frequency.

**Figure 17:**
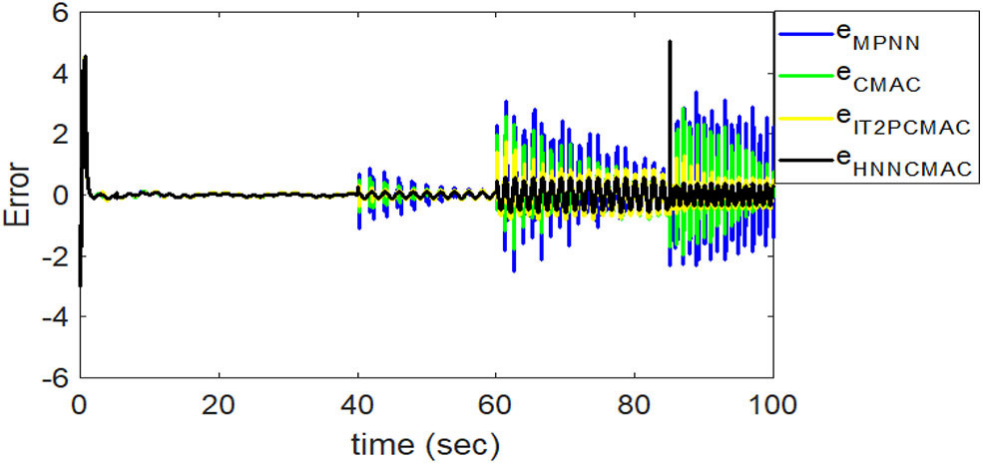
Comparison of tracking errors between proposed HNNCMAC and other control methods for sinusoidal signal reference with varying frequency.

By using the square signal with varying frequency in Equation (35) as the desired trajectory, the reference signal and the system outputs for the time-varying plant are shown in Figure 12. The control signals and tracking errors for this case are shown in Figures 13, 14, respectively. Figure 15 shows the reference signals and system outputs for the time-varying plant when the desired trajectory is the sinusoidal signal with varying frequency in Equation (36). The control signals are plotted in Figure 16, and the tracking errors are plotted in Figure 17. Simulation results for the sinusoidal function reference showed that, at the beginning of the control process, the proposed controller could control the system well, but as frequency increases with time, as well as when the time-varying plant parameters suddenly change, the tracking error also rises due to the controller needing time to adapt to these changes. As shown in Figures 7, 11, 14, 17, there were some rapid variation errors at the time the reference signals or the time-varying plant parameters suddenly changed. However, our proposed controller showed better ability to adapt to these changes, and the tracking error using our proposed HNNCMAC could quickly converge better than other control methods can. The external disturbances and the system uncertainties in this case are chosen as ε(*t*) = 0.8 sin(π*t*) and Δ*y*(*t*) = 0.3 *y*(*t*), respectively. A comparison of the RMSE for the following variable-frequency signal is shown in Table 1.

## Discussions

For this control problems, the HNNCMAC structure had three neurons in the input space, 10 neurons in the hidden space, and two neurons in output space NN. The association space had two layers, each of which with five Gaussian membership functions. The input for the HNNCMAC control system was the output from the sliding hyperplane, its one-step delayed, and its derivatives, *s*(*t*), *s*(*t* − 1), and ṡ(*t*). Term *s*(*t* − 1) is used to obtain more information about the time-varying plants. The initial parameters for the Gaussian function were *m*_11*k*_ = *m*_21*k*_ = *m*_31*k*_ = [−0.5 − 0.3 0 0.3 0.5], *m*_12*k*_ = *m*_22*k*_ = *m*_32*k*_ = [−0.45 − 0.35 − 0.5 0.25 0.45], and σ_*ijk*_ = 0.4. The parameters for the sliding surface were *n* = *3* and λ = 0.2. All learning rates were 0.01, and sampling time was 0.01 s. Using the adaptation laws in Equations (10–23), the controller parameters can be updated online to adapt to the changes in the control system. The examples have demonstrated that our proposed controller can address well the external disturbances and the system uncertainties. The convergence of our proposed controller is guaranteed by Lyapunov stability analysis approach in Equation (29). The average RMSE for all examples between the proposed HNNCMAC, the multilayer perceptron NN (MPNN), the conventional CMAC, the interval type-2 Petri CMAC (IT2PCMAC) (Le et al., 2019), and the type-2 Takagi–Sugeno–Kang fuzzy neural system (T2TSKFNS) (Abiyev and Kaynak, 2010) are shown in Table 1. It is obvious that the proposed controller was using the NN to reduce the inputs for the CMAC; then, the structure of the modified CMAC in our proposed network would be smaller than that of a conventional CMAC. It is more effective when the number of inputs is large. Table 1 shows that the proposed controller has a small computation time than a conventional CMAC, due to our modified CMAC structure was using the NN pre-controller to reduce the computation complexity of the CMAC. Moreover, the NN output used the tangent sigmoid function to limit the output from [−1 1]. Therefore, it is easy to design the network size and the initial membership functions in our modified CMAC controller. As shown in Table 1, the proposed HNNCMAC algorithm could achieve better control performance with the smallest RMSE than other controllers could. In Appendix A, Tables A–D show analysis of the difference between our proposed controller and other controllers using the *t*-Test statistical approach. In all examples, statistical results showed that the *P-value* was lower than the alpha level (α = 0.05). Thus, we can conclude that the RMSE results of our proposed controller had statistically significant difference with other controllers. Therefore, the superiority of the proposed controller was illustrated. Some real-world applications, which have large inputs, can apply this proposed network to reduce the network structure such as medical diagnosis problems, classification problems, image processing problems, etc. Choosing the parameters for the sliding surface affects much of the control performance. This study used the try-and-error approach to obtain suitable parameters. Further studies should investigate the estimation method to estimate these parameters to achieve better control performance.

## Conclusions

This paper proposed an HNNCMAC that is used to control a non-linear dynamic time-varying plant. The main contributions of this study are that it demonstrated a method to control a non-linear dynamic time-varying plant; the HNNCMAC structure uses adaptive laws to adjust parameters online; input range and quantity in the proposed CMAC can be reduced by the NN pre-controller, and it makes it easy to design network size and initial membership functions; the stability of the proposed method is guaranteed by Lyapunov analysis and the numerical-simulation results for controlling a time-varying plant, showing the superiority of the proposed method over existing methods. Moreover, our proposed controller is simple to design and implement, and can be applied to other fields such as system identification, classification, and prediction. Our future work will apply the optimal algorithm to optimize parameters in the sliding surface and learning rates in adaptive laws to achieve better control performance.

## Data Availability Statement

All datasets generated/analyzed for this study are included in the article/Supplementary Material.

## Author Contributions

All authors listed have made a substantial, direct and intellectual contribution to the work, and approved it for publication.

## Conflict of Interest

The authors declare that the research was conducted in the absence of any commercial or financial relationships that could be construed as a potential conflict of interest.
