# Supplementary material for: Hybrid Neural Network Cerebellar Model Articulation Controller Design for Non-linear Dynamic Time-Varying Plants
Source: Front Neurosci. 2020 Jul 28;14:695. doi: 10.3389/fnins.2020.00695 (PMC7399234; doi:10.3389/fnins.2020.00695)
Supplement: Supplementary file 1 [file Data_Sheet_1.docx]

Appendix A

The *t*-Test statistical analysis for the difference between our proposed controller and other controllers.

**Table A.** The statistical test results for Example 1.

|  | ***MPNN*** | ***HNNCMAC*** | ***CMAC*** | ***HNNCMAC*** | ***IT2PCMAC*** | ***HNNCMAC*** |
| --- | --- | --- | --- | --- | --- | --- |
| ***Mean*** | 0.1878 | 0.1215 | 0.1692 | 0.1215 | 0.1408 | 0.1215 |
| ***Variance*** | 2.79E-07 | 4.62E-07 | 5.12E-07 | 4.62E-07 | 5.84E-07 | 4.62E-07 |
| ***df*** | 18 |  | 18 |  | 18 |  |
| ***t_Stat*** | 243.47 |  | 152.88 |  | 59.65 |  |
| ***P_value*** | 4.06E-33 |  | 1.76E-29 |  | 3.86E-22 |  |
| ***CI*** | [0.0657 0.0669] | | [0.0471 0.0484] | | [0.0184 0.0188] | |

**Table B.** The statistical test results for Example 2.

|  | ***MPNN*** | ***HNNCMAC*** | ***CMAC*** | ***HNNCMAC*** | ***IT2PCMAC*** | ***HNNCMAC*** |
| --- | --- | --- | --- | --- | --- | --- |
| ***Mean*** | 0.8679 | 0.6708 | 0.8407 | 0.6708 | 0.7683 | 0.6708 |
| ***Variance*** | 2.03E-07 | 6.09E-07 | 1.80E-07 | 6.09E-07 | 4.42E-07 | 6.09E-07 |
| ***df*** | 18 |  | 18 |  | 18 |  |
| ***t_Stat*** | 691.67 |  | 604.59 |  | 300.67 |  |
| ***P_value*** | 2.80E-41 |  | 3.16E-40 |  | 9.11E-35 |  |
| ***CI*** | [0.1965 0.1977] | | [0.1693 0.1705] | | [0.0968 0.0982] | |

**Table C.** The statistical test results for Example 3 (Square).

|  | ***MPNN*** | ***HNNCMAC*** | ***CMAC*** | ***HNNCMAC*** | ***IT2PCMAC*** | ***HNNCMAC*** |
| --- | --- | --- | --- | --- | --- | --- |
| ***Mean*** | 1.7629 | 1.1644 | 1.7141 | 1.1644 | 1.5297 | 1.1644 |
| ***Variance*** | 1.73E-07 | 2.49E-07 | 1.44E-07 | 2.49E-07 | 2.14E-07 | 2.49E-07 |
| ***df*** | 18 |  | 18 |  | 18 |  |
| ***t_Stat*** | 2911.91 |  | 2772.83 |  | 1696.90 |  |
| ***P_value*** | 1.62E-52 |  | 3.92E-52 |  | 2.70E-48 |  |
| ***CI*** | [0.5981 0.5989] | | [0.5493 0.5501] | | [0.3648 0.3657] | |

**Table D.** The statistical test results for Example 3 (Sinusoidal).

|  | ***MPNN*** | ***HNNCMAC*** | ***CMAC*** | ***HNNCMAC*** | ***IT2PCMAC*** | ***HNNCMAC*** |
| --- | --- | --- | --- | --- | --- | --- |
| ***Mean*** | 0.4901 | 0.3498 | 0.4552 | 0.3498 | 0.4225 | 0.3498 |
| ***Variance*** | 1.83E-07 | 2.28E-07 | 2.02E-07 | 2.28E-07 | 1.82E-07 | 2.28E-07 |
| ***df*** | 18 |  | 18 |  | 18 |  |
| ***t_Stat*** | 691.4390 |  | 508.1526 |  | 358.9424 |  |
| ***P_value*** | 2.82E-41 |  | 7.02E-39 |  | 3.76E-36 |  |
| ***CI*** | [0.1399 0.1407] | | [0.1050 0.1058] | | [0.0723 0.0731] | |

***df*:** degrees of freedom; ***t_Stat***: difference represented in standard-error units; ***P-value***: probability value; ***CI***: confidence interval.
